# Eurypsychrophilic acidophiles: From (meta)genomes to low-temperature biotechnologies

**DOI:** 10.3389/fmicb.2023.1149903

**Published:** 2023-03-15

**Authors:** Mark Dopson, Carolina González-Rosales, David S. Holmes, Nadia Mykytczuk

**Affiliations:** ^1^Centre for Ecology and Evolution in Microbial Model Systems (EEMiS), Linnaeus University, Kalmar, Sweden; ^2^Center for Bioinformatics and Genome Biology, Centro Ciencia & Vida, Fundación Ciencia & Vida, Santiago, Chile; ^3^Facultad de Medicina y Ciencia, Universidad San Sebastian, Santiago, Chile; ^4^Goodman School of Mines, Laurentian University, Sudbury, ON, Canada

**Keywords:** acidic (microbial) environments, astrobiology, bio-applications, polyextremophile, snowball earth, stenopsychrophile

## Abstract

Low temperature and acidic environments encompass natural milieus such as acid rock drainage in Antarctica and anthropogenic sites including drained sulfidic sediments in Scandinavia. The microorganisms inhabiting these environments include polyextremophiles that are both extreme acidophiles (defined as having an optimum growth pH < 3), and eurypsychrophiles that grow at low temperatures down to approximately 4°C but have an optimum temperature for growth above 15°C. Eurypsychrophilic acidophiles have important roles in natural biogeochemical cycling on earth and potentially on other planetary bodies and moons along with biotechnological applications in, for instance, low-temperature metal dissolution from metal sulfides. Five low-temperature acidophiles are characterized, namely, *Acidithiobacillus ferriphilus, Acidithiobacillus ferrivorans, Acidithiobacillus ferrooxidans*, “*Ferrovum myxofaciens*,” and *Alicyclobacillus disulfidooxidans*, and their characteristics are reviewed. Our understanding of characterized and environmental eurypsychrophilic acidophiles has been accelerated by the application of “omics” techniques that have aided in revealing adaptations to low pH and temperature that can be synergistic, while other adaptations are potentially antagonistic. The lack of known acidophiles that exclusively grow below 15°C may be due to the antagonistic nature of adaptations in this polyextremophile. In conclusion, this review summarizes the knowledge of eurypsychrophilic acidophiles and places the information in evolutionary, environmental, biotechnological, and exobiology perspectives.

## Introduction

**Figure 1:**
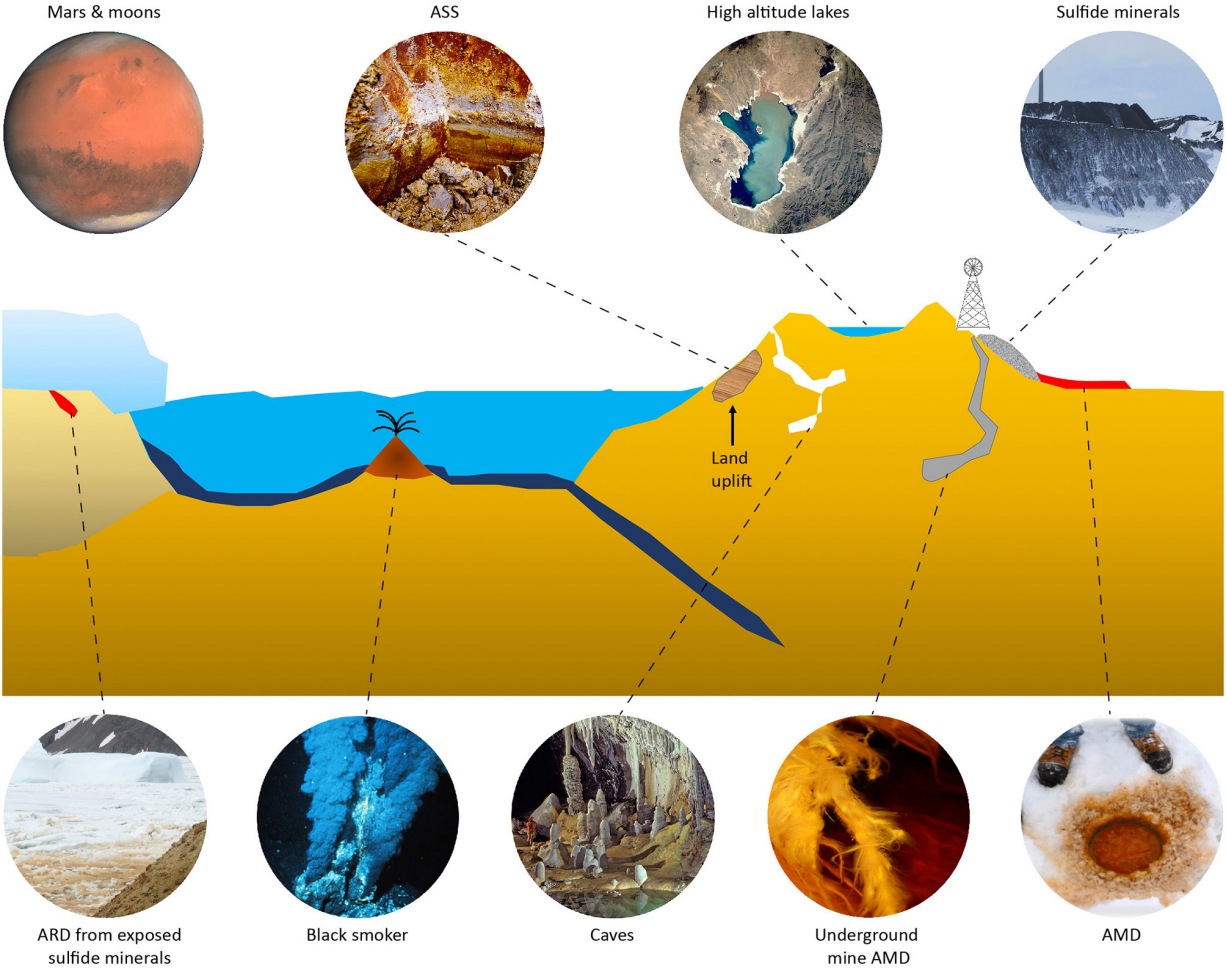
Acidic and low-temperature environments. Clockwise from top left: Mars and moons of Saturn and Jupiter (modified from Wikipedia CC BY-SA 3.0 IGO); boreal AS soil (ASS) created by land uplift (courtesy Eva Högfors-Rönnholm); high altitude lake (modified from Wikipedia public domain image); mining of metal sulfides at, e.g., high altitudes; AMD from under ice sulfide tailings seepage ponds; a low-temperature AMD stream in an underground mine; low-temperature caves (modified photograph by Dave Bunnell from Wikipedia CC BY-SA 2.5); black smokers releasing sulfides (modified from Wikipedia public domain image); and ARD from sulfide minerals, e.g., Antarctica (credit Ángeles Aguilera, Bernhard Dold, and Elena González-Toril), reproduced with permission.

Natural acidic environments include weathered deposits of sulfidic minerals [reviewed in Hedrich and Schippers (2016)] such as in the Iberian pyrite belt where the acidity is generated underground and flows into the Río Tinto (Amils, 2016); volcanic hot pools in, e.g., Hell's Gate (Tikitere), New Zealand (Dunfield et al., 2007); and acid sulfate (AS) soils in Australia (Ling et al., 2015). The generation of anthropogenic acidic environments includes mining and processing of sulfidic ores for the generation of metals including “bioheaps” for copper recovery in the Atacama Desert, Chile (Petersen, 2016). While all the above examples are from warm to hot environments, low-temperature acidic milieus also exist (Figure 1). These include acid rock drainage (ARD) of naturally exposed metal sulfides on, e.g., the South Shetland Islands, Antarctica (Dold et al., 2013); isostatic rebound of Baltic Sea coastal sulfidic sediments that once exposed to air become acid sulfate soils (AS soils) (Boman et al., 2010); and acid mine drainage (AMD) from metal sulfides mines in, e.g., Norway (Johnson et al., 2001).

Microorganisms that inhabit low pH environments are termed “acidophiles,” and they have an optimum pH for growth of < 5, while extreme acidophiles have an optimum pH of < 3 (Johnson and Quatrini, 2016). Acidophilic bacteria obtain energy for growth *via* the oxidation of ferrous iron (Bonnefoy and Holmes, 2012), inorganic sulfur compounds (ISCs) (Dopson and Johnson, 2012), hydrogen (Hedrich and Johnson, 2013), and organic carbon (Johnson and Hallberg, 2009). Extreme acidophiles are found in all three domains of life (Aguilera et al., 2016; Dopson, 2016; Golyshina et al., 2016) although the most extreme are from the Archaea domain that can survive at negative pH values (Tyson et al., 2004). In addition, a large diversity of moderate acidophiles with growth ranges from pH 3 to 7.5, but with optima between pH 4 and 5 inhabit an array of environments including arctic tundra soils (Berestovskaya et al., 2002), *Sphagnum* peat bogs (Dedysh et al., 1998), and sulfidic deep sea sediments (Reysenbach et al., 2006). Characterized extreme acidophiles have growth temperatures ranging from approximately 4°C to >80°C. However, the most well-studied species-type strains have temperature optima ≥30°C including the model species, *Acidithiobacillus ferrooxidans* (Zhang et al., 2018; Moya-Beltrán et al., 2021). In contrast, fewer cold adapted species have been identified, and they are less well studied than their mesophilic and thermophilic counterparts.

Extremophilic microorganisms are used in a variety of biotechnological applications spanning the biomedical, pharmaceutical, industrial, environmental, and agricultural sectors (Raddadi et al., 2015; Banerjee et al., 2021; Verma, 2021). Applications of low-temperature microorganisms have seen numerous successes in the production of cold active proteases, lipases, and cellulases in food production, chemical synthesis, textile industry, pharmaceutical and molecular biology, bioremediation, and novel antimicrobials [reviewed in Cavicchioli et al. (2011) and Siddiqui (2015)]. Acidophiles in particular are exploited for their acid stable enzymes in food/animal feed processing (Sharma et al., 2016), in pharmaceutical delivery (Jensen et al., 2015), in microbial fuel cells (Sulonen et al., 2015; Ni et al., 2016), and in the production of novel biomaterials (Quehenberger et al., 2017). However, the most extensive exploitation of acidophiles is their use in “biomining” of metals such as copper and gold (Roberto and Schippers, 2022) where acidophiles catalyze the conversion of a solid metal sulfide to a soluble metal sulfate (Vera et al., 2022). The same microbes are also used for the remediation of environmentally damaging AMD that results from the extraction of metals (Nancucheo et al., 2017). However, as most biomining is carried out in warm countries and that pyrite/pyrrhotite oxidation is exothermic resulting in bioheaps reaching temperatures up to 90°C (Puhakka et al., 2007), investigation of low-temperature acidophiles in biotechnologies lags behind studies of mesophiles and thermophiles. Some mines and their corresponding impacted environments are located in the Polar Regions or high altitudes that are predominantly cold (e.g., Elberling et al., 2000). Further expansion of these biotechnologies requires a deeper understanding of low-temperature acidophiles.

Our interest in this review is in the particular niches occupied by extremely acidophilic prokaryotes that are also capable of growth at colder temperatures. To date, this only encompasses bacteria, as although cold-adapted archaea have been identified (Cavicchioli, 2006; Siddiqui et al., 2013), no low-temperature acidophilic Archaea have been described. It is unknown if this absence is due to sampling bias, and they remain to be discovered, or if they do not exist due to unidentified biological constraints. The fact that acidophiles are active at low temperatures has been known for many years since strains were isolated in the 1980s and the activities of these populations were observed in the environment down to 0°C (Langdahl and Ingvorsen, 1997).

Several terms have been formulated for low-temperature microorganisms including psychrotolerant and psychrotrophs with various definitions. A further term is “eurypsychrophiles” for microbes with a wide temperature tolerance that are able to grow at temperatures above and below 15°C but with a maximum growth temperature of 30°C (Bakermans and Nealson, 2004). In this review, we have used eurypsychrophiles but also included some strains with a maximum growth temperature slightly above Bakermans and Nealson (2004) cutoff. Although eurypsychrophilic acidophiles are still poorly understood, new data are emerging on this valuable and untapped niche with a range of potential biotechnological applications as well as their use as models for potential past or extant life on astrobiological targets.

## Strategies to cope with acid and low temperature

To maintain a near pH neutral cytoplasm, acidophiles have a range of mechanisms defined as the “first and second lines of defense” (Vergara et al., 2020) similar to those found in neutrophilic microbes as well as several unique homeostasis strategies [reviewed in Slonczewski et al. (2009) and Zammit and Watkin (2016)]. Several studies have also investigated the evolution of acidophiles by the acquisition of genes coding for the first and second line of defense (Vergara et al., 2020; Boase et al., 2022; González-Rosales et al., 2022). The unique strategies include an inside positive membrane potential (i.e., reversed compared to neutrophiles) suggested to be generated by potassium or sodium ions that create an electrochemical barrier to proton influx (Buetti-Dinh et al., 2016; Neira et al., 2022). A second unique strategy is that acidophiles can have a rigid and impermeable membrane that is highly resistant to the influx of protons (van de Vossenberg et al., 1998). The membrane is also modified in acidophiles *via* membrane proteins with adaptations that impede proton permeability, such as a unique Omp40 porin protein in *At. ferrooxidans* (Guiliani and Jerez, 2000). Mechanisms of pH homeostasis that are shared with neutrophilic microorganisms include cytoplasmic buffering that in the short term can ameliorate acidification of the cytoplasm (Parro et al., 2007; Vergara et al., 2020). In addition, primary and secondary pumps exist that can remove excess cytoplasmic protons (González et al., 2014). Acidophiles also have the capacity to modify and repair DNA as a general response to damage (Parro et al., 2007), and acidophiles may have a streamlined genome in response to their acidic growth pH (Cortez et al., 2022). Finally, the “*Fervidacidithiobacillus caldus”* (*ex-Acidithiobacillus caldus*) ferric uptake regulator (Fur) is important in acid resistance and shock by regulating genes involved in, e.g., key genes of certain cellular activities, such as iron transport, biofilm formation, and ISC metabolism (Chen et al., 2020).

Microbes that live at cold temperatures must deal with several biochemical and physiological challenges. These include reduced water activity, membrane fluidity (Bowman, 2008; Shivaji and Prakash, 2010), protein folding and activity (Feller, 2007; Collins et al., 2008; Metpally and Reddy, 2009), and enzyme reaction rates (Feller, 2007; Collins et al., 2008) along with increased osmotic pressure (Bowman, 2008) and risks of freezing (Kawahara, 2008) that are reviewed in Casanueva et al. (2010), De Maayer et al. (2014), and Dhaulaniya et al. (2019). Regarding eurypsychrophiles in general, reviews have highlighted key features of cold adaptation across a diversity of microorganisms (Margesin et al., 2008; Margesin and Miteva, 2011; Tribelli and Lopez, 2018). These can include increased enzyme flexibility through decreased core hydrophobicity, weaker interactions between subunits, fewer disulfide bridges, along with increased catalytic efficiency between the protein and substrate (Marx et al., 2007; Cavicchioli et al., 2011; Badieyan et al., 2012; Lian et al., 2015). Membranes of psychrophilic bacteria maintain their fluidity and phase through various mechanisms. These include increased amounts of unsaturated fatty acids, increased branched fatty acids, favoring higher *cis*/*trans* unsaturated fatty acid ratios, the synthesis of fatty acids with shorter acyl chain length, and larger or differently charged polar head groups (Los and Murata, 2004; Shivaji and Prakash, 2010; Králová, 2017). In both bacterial and archaeal membranes, the destabilization caused by membrane lipid alterations is commonly offset with an increase in pigments or carotenoids that provide added stability within the liquid-crystalline membrane state at colder temperatures (Chattopadhyay et al., 1997). The response to cold shock also triggers adaptive mechanisms. These include the induction of cold shock proteins and increased expression of chaperones to assist with RNA stability, protein folding, and enzymatic activities, along with increased compatible solute synthesis and/or uptake, extracellular polysaccharide synthesis, or ice interacting proteins (Junge et al., 2006; Phadtare and Inouye, 2008; Jung et al., 2010, 2018; Galleguillos et al., 2018; Kumar et al., 2020).

## Eurypsychrophilic acidophiles

### Characterized eurypsychrophilic acidophiles

**Figure 2:**
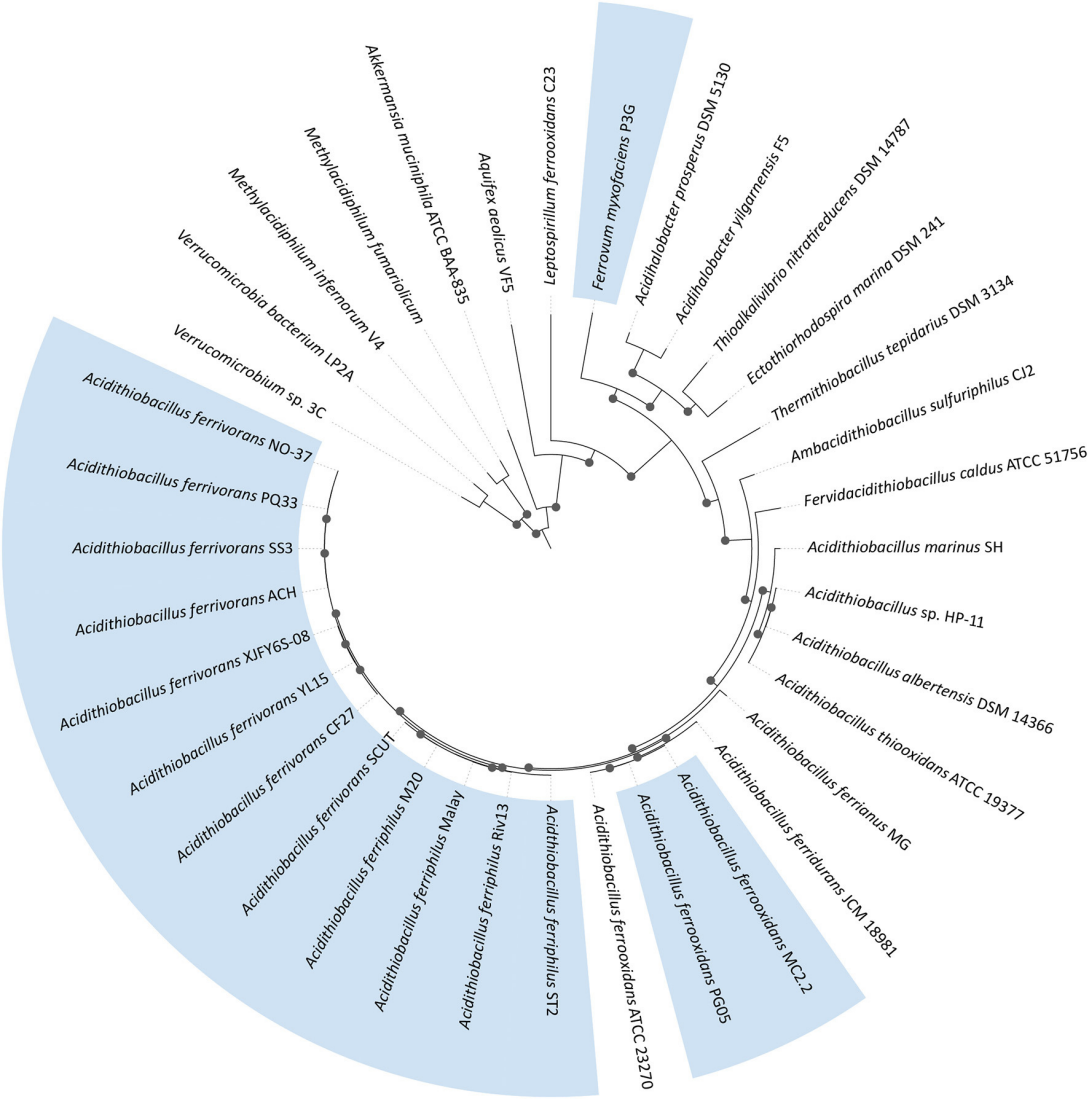
Maximum likelihood phylogenetic tree of eurypsychrophilic acidophiles (in blue) plus relatives. The tree was constructed using 31 conserved proteins (Ciccarelli et al., 2006) using 1,000 replicates. Bootstrap values of ≥60% are represented by black dots on the nodes. The phylogenomic tree was visualized with iTOL6 (Letunic and Bork, 2021).

The characterized eurypsychrophilic acidophiles are from the Proteobacteria (*Acidithiobacillus ferrivorans, Acidithiobacillus ferriphilus, Acidithiobacillus ferrooxidans*, and “*Ferrovum myxofaciens*”) and the Firmicutes (*Alicyclobacillus disulfidooxidans*; no genome sequence available) with their general characteristics summarized in Table 1 and their phylogenetic relationships represented in Figure 2.

**Table 1:** Selected characteristics of eurypsychrophilic acidophile species along with their genome availability.

**Species**	**Strain**	**Selected growth characteristics**	**Temperature range**	**Genome state**	**Genome accession**
*At. ferrivorans*	PQ33	Facultative anaerobe; fixes C and N; oxidizes Fe^2+^, ISCs, and H_2_; optimum pH 2.5;	5–24°C	Draft	CP021414.1 and CP021415.1
	SS3	Oxidizes Fe^2+^ and ISCs; grows at 6°C with optimum 22°C; growth optimum pH 1.9	5–30°C	Complete	CP002985.1
	YL15	Grown at pH of 2.0	6–28°C	Draft	MASQ01000000
	CF27	Notably forms large amount of extracellular polymeric substances during biofilm formation	< 10–30°C	Complete	LT841305.1 and LT841306.1
	XJFY6S-08	Grown aerobically at pH 1.75	5–28°C	Complete	CP059488.1
	ACH	Oxidizes Fe^2+^, ISCs, and FeS_2_; attaches to mineral at 4°C	10–28°C	Draft	JAAZUD000000000
	NO-37	Fixes C and N; oxidizes Fe^2+^, ISCs, and H_2_; optimum of 27°C to 32°C; pH optimum pH 2.5 (range pH 1.9 to 3.4)	4–37°C	Draft	JAAOMR000000000
*At. ferriphilus*	M20	Facultative anaerobe; fixes C; oxidizes Fe^2+^ and ISCs; optimum temperature of 30°C and pH of 2.0	5–33°C	Draft	JAAZTY000000000
	Malay	Facultative anaerobe; fixes carbon; oxidizes Fe^2+^ and ISCs; optimum pH ~2.0; optimum temperature ~30°C	5–33°C	Draft	JAAOMQ000000000
	Riv13		10–33°C	Draft	JAAZUB000000000
	ST2	Grows in high salt concentrations	5–33°C	Draft	JAAZUC000000000
	SCUT-1	Oxidizes Fe^2+^, ISCs, and FeS_2_	22.1–30°C	Draft	NKQV00000000
*At. ferrooxidans*	PG05	Growth optima ≈20°C; contain polyphosphate granules	5–33°C	Draft	JAEQBE000000000
	MC2.2	Growth optima ≈20°C; contain polyphosphate granules	5–33°C	Draft	JAEQBG000000000
“*Ferrovum myxofaciens*”	P3G	Aerobe; fixes carbon and nitrogen; oxidizes Fe^2+^; optimum pH 3.0 and temperature 32°C	4–36°C	Draft	JPOQ00000000
*Alb. disulfidooxidans*	SD-11	Mixotroph; oxidizes S^0^ and FeS_2_; optimum pH 1.5 range 0.5 to 6	4–40°C	—	Not available

*At., Acidithiobacillus*; *Alb., Alicyclobacillus*; and ISCs, inorganic sulfur compounds.

The first eurypsychrophilic acidophile to be formally described was *At. ferrivorans* NO-37^T^ (Hallberg et al., 2010). The type strain was isolated from pH 2.7 to 3.7 AMD waters at an abandoned copper mine in Norway where temperatures are below freezing for approximately 7 months of the year (Johnson et al., 2001). *At*.*ferrivorans*^T^ is a Gram-negative facultative anaerobe that fixes carbon dioxide and nitrogen for growth with ferrous iron and ISCs as electron donors. *At*.*ferrivorans*^T^ grows at 4°C although its optimum growth temperature is 27°C to 32°C, and it has an optimum pH of 2.5 (growth range at least pH 1.9 to 3.4). Due to the type strain's growth at 32°C, it does not match the criterion used for eurypsychrophilic acidophiles (Bakermans and Nealson, 2004). Other isolates of *At. ferrivorans* comprise strains Peru6, OP14, and CF27 included in the analysis of the type strain (Hallberg et al., 2010), and a notable feature of strain CF27 is its formation of large amounts of extracellular polymeric substances during biofilm formation on mineral grains (Talla et al., 2014). *At. ferrivorans* SS3 was isolated from a sulfidic mineral mining site and is characterized as having a lower temperature optimum at 22°C and growing as low as 6°C using ferrous iron (Kupka et al., 2007) and ISCs (Kupka et al., 2009). Strain PQ33 that was isolated from an altitude of 4,621 m above sea level in Peru has a doubling time of 66.6 ± 5.1 h at pH 1.6 (Ccorahua-Santo et al., 2017). Strain YL15 was also isolated at altitude (4,600 m above sea level) from AMD at a copper mine in Tibet, China and grows on ferrous iron at both 6°C and 28°C (Peng et al., 2017). In addition, isolates PA-5, PA-8, and PA-20 were obtained from AMD in Balya, Turkey by culturing on ferrous iron containing plates (Aytar et al., 2015). Finally, *At. ferrivorans* ACH was isolated from the Aroma River in the Chilean Altiplano that has an average annual temperature of < 5°C, grows on ferrous iron, ISCs, and pyrite as electron donors, and is able to attach to pyrite mineral at 4°C (Barahona et al., 2014).

Eleven strains of facultative anaerobic, ferrous iron and ISC oxidizing, autotrophic *At. ferriphilus* are characterized in the species description, and all are able to grow at 30°C (Falagan and Johnson, 2016). All of the strains were able to grow at 10°C (although some slowly) and three at 5°C including the type strain, classifying them as a eurypsychrophilic acidophiles. *At. ferriphilus* strains have been identified from, e.g., mine environments, while strains ST2 and KCT10 grew at salt concentrations similar to that of seawater.

A further eurypsychrophilic acidophile from the *Acidithiobacillus* genus is represented by two strains (PG05 and MC2.2) of *At. ferrooxidans* isolated from an old mine in southern Chile and King George Island, Antarctica, respectively (Muñoz-Villagrán et al., 2022). In addition, both strains, but especially PG05, have cellular electron dense areas indicative of polyphosphate granules that act in the response to stress conditions.

The fourth characterized eurypsychrophilic acidophile is the Betaproteobacteria “*F. myxofaciens*” (reclassified within the Gammaproteobacteria according to the GTDB database; Chaumeil et al., 2019). The proposed type strain, P3G, was cultured from AMD emanating from the Mynydd Parys, Wales (Johnson et al., 2014) where it dominated the streamer-type biofilms at this (Kay et al., 2013) and another mine site (Kimura et al., 2011). “*F. myxofaciens*” has a pH optimum of approximately 3.0, grows optimally at 30°C to 32°C, and exclusively uses ferrous iron and oxygen as electron donors and acceptors, respectively. “*F. myxofaciens*” P3G fixes both carbon dioxide and nitrogen to support the production of large amounts of extracellular polymeric substances that bind the cells together in streamer-like biofilms and to solid surfaces (Johnson et al., 2014).

A fifth eurypsychrophilic acidophile capable of not only sulfur oxidation but also heterotrophic metabolism is *Alb. disulfidooxidans* 51911^T^ (originally described as *Sulfobacillus disulfidooxidans*). This strain was isolated from wastewater sludge, has an optimal pH between 1.5 and 2.5, and a broad temperature range of 4°C to 40°C (Dufresne et al., 1996; Karavaiko et al., 2005).

### Environmental strains and 16S *rRNA* gene sequences

Studies of mine tailings from Canadian arctic regions suggest microbial activity below 0°C (Meldrum et al., 2001; Elberling, 2005) with a potential explanation of solutes maintaining liquid water below 0°C or that local temperatures were higher due to exothermic reactions, especially if the cells were cocooned in biofilms. The microbial community in several low-temperature acidic environments has been characterized by traditional culturing and molecular 16S *rRNA* gene sequencing. In most cases, the identification of particular species did not always coincide with viability or activity measurements at cold temperatures. However, repeated observations indicate a diversity of eurypsychrophilic acidophiles in these environments. Some of the earliest reports of cold-tolerant acidophiles from AMD environments are reported as existing in Scandinavia and Canada, with their taxonomy determined to be *Thiobacillus ferrooxidans*-like or *At. ferrooxidans-*like (Ferroni et al., 1986; Ahonen and Tuovinen, 1989). Of these, *At. ferrivorans* strains D2, D6, and D7 were isolated from the Denison Uranium mines in Ontario, Canada (Ferroni et al., 1986; Berthelot et al., 1993). They were characterized as having a growth temperature optimum of 25°C and growing as low as 2°C using ferrous iron (Leduc et al., 1993) with a growth range of at least pH 1.5 to 3.5 and an optimum of 2.1 (Mykytczuk et al., 2010b). These strains were also characterized for high metal tolerance that was maintained even when grown at colder temperatures with minimum inhibiting concentrations of 150 mM copper, 50 mM nickel, and 2.0 mM uranium (Leduc et al., 1997; Mykytczuk et al., 2011b).

In addition to the reports above, other studies used cultivation, molecular tools, and microscopy to observe diverse communities from cold and acidic environments. These include several descriptions of *At. ferrooxidans* and *At. ferrivorans* from Scandinavian mine sites (Kock and Schippers, 2008; Kupka et al., 2009); a 7.9 to 13.9°C acidic drainage system in Argentina containing a consortium including *At. ferrivorans* (Bernardelli et al., 2021); ice covered AMD ponds (Auld et al., 2017); *At. ferrivorans* and *Thiobacillus plumbophilus* from natural ARD in the South Shetland Islands, Antarctica (Dold et al., 2013); and mixed communities of chemolithotrophs plus chemoorganotrophs in mine waste sites, Ontario Canada (Asemaninejad et al., 2020). Many of these reports also include detection of other acidophiles including chemolithotrophs such as *Leptospirillum ferrooxidans* and *Ferrimicrobium* spp. from a coal mine in Svalbard, Norway (Garcia-Moyano et al., 2015) along with *L. ferrooxidans* and *Sulfobacillus* spp. from a tailings dump in Kristineberg, Sweden (Kock and Schippers, 2008). Heterotrophic acidophiles were also detected in these systems including *Acidiphilium* and *Alicyclobacillus* spp. (Auld et al., 2017), *Acidisphaera* spp., and *Acidobacterium* spp. (Garcia-Moyano et al., 2015).

AS soils are generated when parent sulfidic sediments are exposed to air, and the reduced sediment is oxidized to release acidic, metal-laden solutions (Karimian et al., 2018). Boreal AS soils typically reach pH 3 to 5 and therefore contain a mixture of extreme and moderate acidophiles as well as a higher relative proportion of heterotrophic vs. autotrophic acidophiles compared to low organic carbon containing AMD (Högfors-Rönnholm et al., 2018). Samples taken from the oxidized layer in a Finnish AS soil contained many 16S *rRNA* gene sequences that aligned most closely to acidophiles found in AMD such as the ferrous iron and ISC-oxidizing *At. ferrooxidans*, the ferrous iron-oxidizing archaeon *Ferroplasma acidiphilum*, the acid-tolerant *Rhodanobacter* genus, and an uncultured *Acidimicrobiaceae* clone from an extremely acidic environment (Wu et al., 2013, 2015; Högfors-Rönnholm et al., 2018). However, if the parent sulfidic sediment is rapidly oxidized in the laboratory by active aeration, the pH level is decreased to 3, and sequences more similar to typical AMD environments are selected such as the low-temperature acidophile, *At. ferrivorans* (Wu et al., 2015). Finally, if metal sulfide-rich river sediment were to be dredged and the sediment is exposed to atmospheric oxygen, rapid oxidation occurs and representatives of the *Acidithiobacillus, Gallionella, Sulfuricurvum*, and *Sulfurimonas* genera become dominant (Johnson et al., 2022).

### Growth substrates and oxidation rates

As noted in the characterization section above, both *At. ferrivorans* and “*F. myxofaciens*” can use ferrous iron as an electron donor (Hallberg et al., 2010; Johnson et al., 2014). In addition, ferrous iron appears to be *At. ferrivorans* SS3 preferred substrate as it is preferentially oxidized over ISCs (Liljeqvist et al., 2013). The rate constant for strain SS3 ferrous oxidation at 5°C is 0.0162–0.0104 h^−1^ dependent on the initial pH of the culture. As for other strains, the *At. ferrivorans* PQ33 ferrous oxidation rate is higher at 24°C compared to 5°C when it has a doubling time of 66.6 ± 5.1 h at pH 1.6 (Ccorahua-Santo et al., 2017). Eurypsychrophilic *At. ferrooxidans* strains oxidized ferrous iron and grew down to 5°C with doubling times between 12.8 and 14.1 h but had optimum growth rates at 20°C while still having a high growth rate at 37°C (Muñoz-Villagrán et al., 2022). Ferrous iron oxidation rates for “*F. myxofaciens*” peaked at 30°C, a value 12.5-fold higher than the corresponding rate at 5°C (Johnson et al., 2014). Ferrous iron is also a preferred substrate over ISCs for the *At. ferrivorans* D-strains (D2, D6, and D7; previously identified as *At. ferrooxidans*), and they have doubling rates of 253–306 ± 22 h when grown at 2°C and at pH 2.1 and 87–88 ± 1.5 h at 5°C (Leduc et al., 1993; Mykytczuk et al., 2011a). The combination of cold and sub- or supra-optimal pH increases these doubling rates, but are comparably longer than under pH stress alone (i.e., growth rates of 25–38 ± 3.3 h at pH 1.5 in ferrous iron media) (Mykytczuk et al., 2010a).

*At. ferrivorans* NO-37^T^ oxidizes sulfide, tetrathionate, thiosulfate, and elemental sulfur (Hallberg et al., 2010). ISC oxidation is most extensively investigated in *At. ferrivorans* SS3 with extended lag phases with tetrathionate as an electron donor at 5°C (Kupka et al., 2009). Temperature optima for tetrathionate and elemental sulfur were 25°C and 20°C, respectively. The *At. ferrivorans* D2, D6, and D7 strains also grow on various ISCs including sulfide, sulfite, thiosulfate, and elemental sulfur, but in all cases, they were grown at temperatures between 20°C and 25°C but not at lower temperatures (Berthelot et al., 1993; Leduc et al., 1993).

In the environment, dissimilatory ferric iron reduction is an important electron acceptor for the oxidation of organic and inorganic electron donors and is a driver in the iron cycle (Bird et al., 2011). Mesophilic and moderately thermophilic acidophiles carry out dissimilatory reduction of both soluble ferric iron and solid ferric minerals (Bridge and Johnson, 1998, 2000; Osorio et al., 2013; Malik and Hedrich, 2022), and a biotechnological process has been proposed that exploits this ability to extract, e.g., nickel from laterites (du Plessis et al., 2011; Hallberg et al., 2011). *At. ferrivorans* strains NO-37^T^, Peru6, and CF27 anaerobically oxidize elemental sulfur as electron donors coupled to the reduction of ferric iron (Hallberg et al., 2011). In addition, *At. ferrivorans* SS3 coupled elemental sulfur to ferric iron reduction even in the presence of molecular oxygen (Kupka et al., 2009).

*Alb. disulfidooxidans* 51911^T^ metabolizes a wide range of organic carbon compounds including starch, glycogen, and glucose (Dufresne et al., 1996). Its ability to oxidize organic carbon, and in particular, organic acids places this species at a competitive advantage as it is able to reduce the concentration of these acids that are toxic to extreme acidophiles *via* acidification of the cytoplasm leading to cell death (Alexander et al., 1987).

## Genomics and post genomics

Acidophiles have proven to be recalcitrant to analysis *via* traditional molecular biology tools, and only a few studies have succeeded such as the generation of *At. ferrooxidans* mutants (Jung et al., 2021); instead, acidophiles have been extensively studied by “omics” technologies. Consequently, there are approximately 600 finished or permanent draft acidophile genomes, 184 extreme acidophiles (optimum pH ≤ 3.0) genomes of which 15 are from eurypsychrophilic acidophiles (Neira et al., 2020). These studies allow the identification of genetic potential and RNA transcript-based gene activities of these populations.

### Eurypsychrophilic acidophile genomes and post genomics

Several genomes of eurypsychrophilic acidophilic bacteria have been published (Table 1) including seven *At. ferrivorans* strains, namely, SS3 (Liljeqvist et al., 2011b), CF27 (Talla et al., 2013), PQ33 (Ccorahua-Santo et al., 2017), YL15 (Peng et al., 2017), ACH (Barahona et al., 2020), NO-37 (Moya-Beltrán et al., 2021), and XJFY6S-08 (Zhao et al., 2021). The *At. ferrivorans* SS3 genome codes for genes for carbon dioxide fixation *via* the Calvin-Benson-Bassham (CBB) cycle; nitrogen metabolism; ferrous iron oxidation; and ISC metabolism including the SOX oxidation complex (*soxYZ-hypB*) and a sulfur oxygenase:reductase *sor* (Liljeqvist et al., 2011b). The *At. ferrivorans* SS3 ISC pathway was also identified by transcriptomics that revealed RNA transcripts for a thiosulfate quinone oxidoreductase (*doxDA*), heterodisulfide reductase (*hdr*) for elemental sulfur metabolism, and electron transport complexes for energy conservation (Christel et al., 2016a). The *At. ferrivorans* CF27 genome was also analyzed and found to code for an *iro* gene absent in *At. ferrooxidans* suggesting an alternative pathway for ferrous iron oxidation (Talla et al., 2013). Similar to strain SS3, genes involved in ISC oxidation were identified including sulfide oxidation (*sqr*), tetrathionate hydrolase (*tetH*), and sulfite oxidation (*sat* and *cytC*). The strain CF27 genome also contains genes encoding fucose biosynthesis (*fcl*) and glycosylation of surface layer glycoprotein that could explain the strains propensity to form gelatinous macroscopic biofilms (Talla et al., 2014). Finally for strain CF27, the genome harbors 51 genomic islands coding for genes associated with, e.g., chemotaxis, motility *via* flagella, and biofilm synthesis (Tran et al., 2017). Strain YL15 was isolated from an AMD environment, and its genome contains genes for metal resistance such as the *mer* operon for mercury, *arsRABCD* genes for arsenic, and *copB* plus *cusCBA* for copper (Peng et al., 2017). Microorganisms at high altitudes such as the alpine region from where strain YL15 was isolated are exposed to increased levels of ultraviolet radiation and the strain's genome coded for mycosporine-like amino acid production that combat this along with reactive oxygen scavenging systems that are generated by the radiation (Peng et al., 2017). In addition, the *At. ferrivorans* PQ33 genome harbors a genomic island containing a *rusB* gene (Ccorahua-Santo et al., 2017). Finally, strain *At. ferrivorans* XJFY6S-08, also from acid mine drainage, contains genes for metal assimilation plus resistance systems and DNA repair (Zhao et al., 2021), while strain ACH was analyzed for its ability to grow in high copper concentrations (up to 400 mM) and the corresponding resistance determinants included the *cop* and *cus* systems (Barahona et al., 2020).

The *At. ferriphilus* M20 and Riv13 genomes were sequenced during a large-scale characterization of the Acidithiobacillia class with the M20 strain containing genes coding for ferrous iron oxidation including *rus, cyc2*, and *petABC*; ISC metabolisms such as *sqr*, sulfur dioxygenase (*sdo*), *hdrABC, tetH*, and *doxDA*; and hydrogenases I-IV (Moya-Beltrán et al., 2021). *At. ferriphilus* SCUT-1 genome sequencing revealed genes involved in ferrous iron oxidation including a *rus* gene cluster (Fan et al., 2018). In addition, genes encoding ISC oxidation included *sqr, tetH, tqo, soxYZB* genes, and *hdrABC* that feed electrons to *aa*_3_ and *bo*_3_ electron acceptors. Finally, the *At. ferriphilus* Malay and ST2 genomes contain genes coding for ferrous iron and ISC metabolism (Moya-Beltrán et al., 2014).

The “*F. myxofaciens*” P3G genome also contains genes coding for the complete CBB cycle including carboxysome formation along with *nif* genes for the nitrogenase complex for carbon dioxide and nitrogen fixation, respectively (Moya-Beltrán et al., 2014). “*F. myxofaciens*” genes for ferrous iron oxidation are similar to the well-studied *At. ferrooxidans* system with an outer membrane *c*-type cytochrome connected with a chain of cytochromes with the exception of the cytochrome oxidase terminal electron acceptor that is absent (Quatrini et al., 2009). Obligate ferrous iron-oxidizing acidophile species also carry out “up-hill” electron transport to generate reducing power, and genes coding for the integral *bc*_1_ complex were also identified (Moya-Beltrán et al., 2014). Finally, despite the type-strain being described as non-motile, genes coding for chemotaxis and flagella formation are present.

### Low-temperature metagenomes and metatranscriptomes

Community DNA analysis of sulfide mineral mining environments suggests that the presence of eurypsychrophilic acidophiles and their metagenome-assembled genomes (MAGs) have been analyzed. Included in this section are community studies from acidic and low-temperature environments that match the criteria of ≤ pH 3.1 and 15°C with the caveat that average environmental pH and temperatures are insufficient to prove the presence of eurypsychrophilic acidophiles.

Community DNA was extracted from a streamer-type biofilm in a 6°C to 10°C AMD stream in northern Sweden (Liljeqvist et al., 2015). Both the biofilm and planktonic cells were dominated by an *At. ferrivorans* strain along with other *Acidithiobacillus, Acidobacteria*-like, and *Gallionellaceae*-like populations. A comparison of the *At. ferrivorans* SS3 genome to the *At. ferrivorans*-like MAG identified metagenomic islands enriched with mobile elements and containing genes coding for metal resistance related to the high concentrations of metals in the AMD stream from where the *At. ferrivorans*-like strain was assembled (González et al., 2014).

A cold AMD stream (6.5°C and pH 2.65) was also sampled for community metagenomics from the Sherlovaya Gora mine, Eastern Siberia, Russia. The community was dominated by an uncultured ferrous iron and ISC-oxidizing *Candidatus* Gallionella acididurans species with lesser contributions from the genera *Thiobacillus, Acidobacterium, Acidisphaera*, and *Acidithiobacillus* (Kadnikov et al., 2016). Four additional Chinese AMD sites were analyzed by community “omics” with RNA transcripts suggesting *At. ferrivorans* populations were highly active in three of the four sites with a broad range of functions (Chen et al., 2015). For instance, RNA transcripts were detected for the majority of *At. ferrivorans* genes for ferrous iron and ISC oxidation along with nitrogen fixation. A further Chinese AMD study was used to assemble a 79% complete *Ferrovum* genome that had a relative abundance of >90% (Hua et al., 2015). RNA transcripts suggest this population to be fixing carbon dioxide but not nitrogen along with the highest number of RNA transcripts coding for oxidative phosphorylation.

A mine water treatment plant was analyzed by metagenomics from which a nearly complete genome of *Ferrovum* strain JA12 was assembled and suggested to be a second species alongside “*F. myxofaciens*” (Ullrich et al., 2016b). The JA12 genome was small (2.70 Mbp) but encoded *CBB cycle* genes for carbon dioxide fixation; assimilation of nitrogen from ammonium, nitrate, and urea; ferrous iron oxidation *via* a Cyc2-like protein; and both up- and downhill electron transport chains. JA12 adaptations to its environment include metal resistance genes such as *copA* and *arsC* (Ullrich et al., 2016b). Two further *Ferrovum* strains (Z-31 and PN-J185) were assembled from the same mine water treatment pilot plant as JA12 in Nochten, Germany, with strain PN-J185 suggested to represent a third *Ferrovum* species (Ullrich et al., 2016a). Comparative genomics of the three strains together with the P3G type strain genome revealed distinctive metabolic profiles related to motility, chemotaxis, nitrogen fixation, and biofilm generation. An additional 8.1°C to 14.6°C AMD site in Cabin Branch, USA, is dominated by 16S *rRNA* gene amplicon sequences most closely related to “*F. myxofaciens*” and metagenome sequencing resulted in two nearly complete *Ferrovum* MAGs that also differed according to genes coding for motility and chemotaxis along with, e.g., nitrogen assimilation (Grettenberger et al., 2020). In addition to the *Ferrovum* MAGs, assembled genomes related to, e.g., the *Gallionella* and *Acidocella* genera along with a Thermoplasmata archaeon were identified.

Finally, metagenomes from a low-temperature boreal acid sulfate soil situated in Vasa, Finland showed that the metal sulfide oxidation of the parent reduced sediment was dominated by MAGs aligning with the acid-tolerant or moderately acidophilic iron-oxidizing *Gallionella* and sulfur-metabolizing *Sulfuricella* genera (Högfors-Rönnholm et al., 2022). In contrast to most (but not all) sequenced *Gallionella* genomes, the acid sulfate soil MAGs were suggested to also encode ISC oxidation.

## Eurypsychrophilic acidophile adaptations

**Figure 3:**
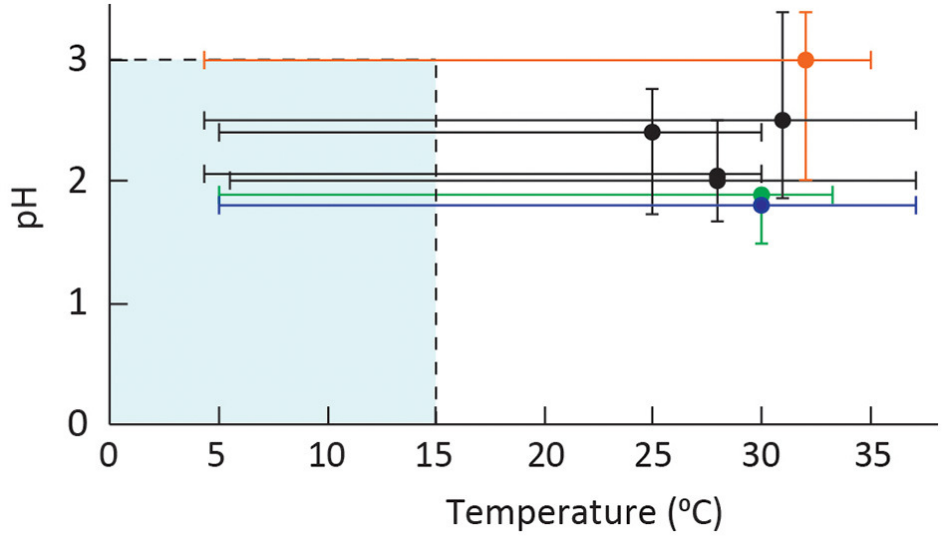
Plot of pH vs. temperature optima and ranges of characterized eurypsychrophilic acidophiles with the limits of low-temperature growth and extreme acidophiles are shaded in blue. The species are *A. ferrivorans* strains (all in black) from top to bottom NO-37, CF27, SS3, and ACH; *At. ferrooxidans* PG05 and MC2.2 represented as a single line (blue); “*F. myxofaciens*” P3G (orange); and *A. ferriphilus* M20 (green). The strains with an optimum pH of 2.5 were slightly adjusted for visual clarity.

**Figure 4:**
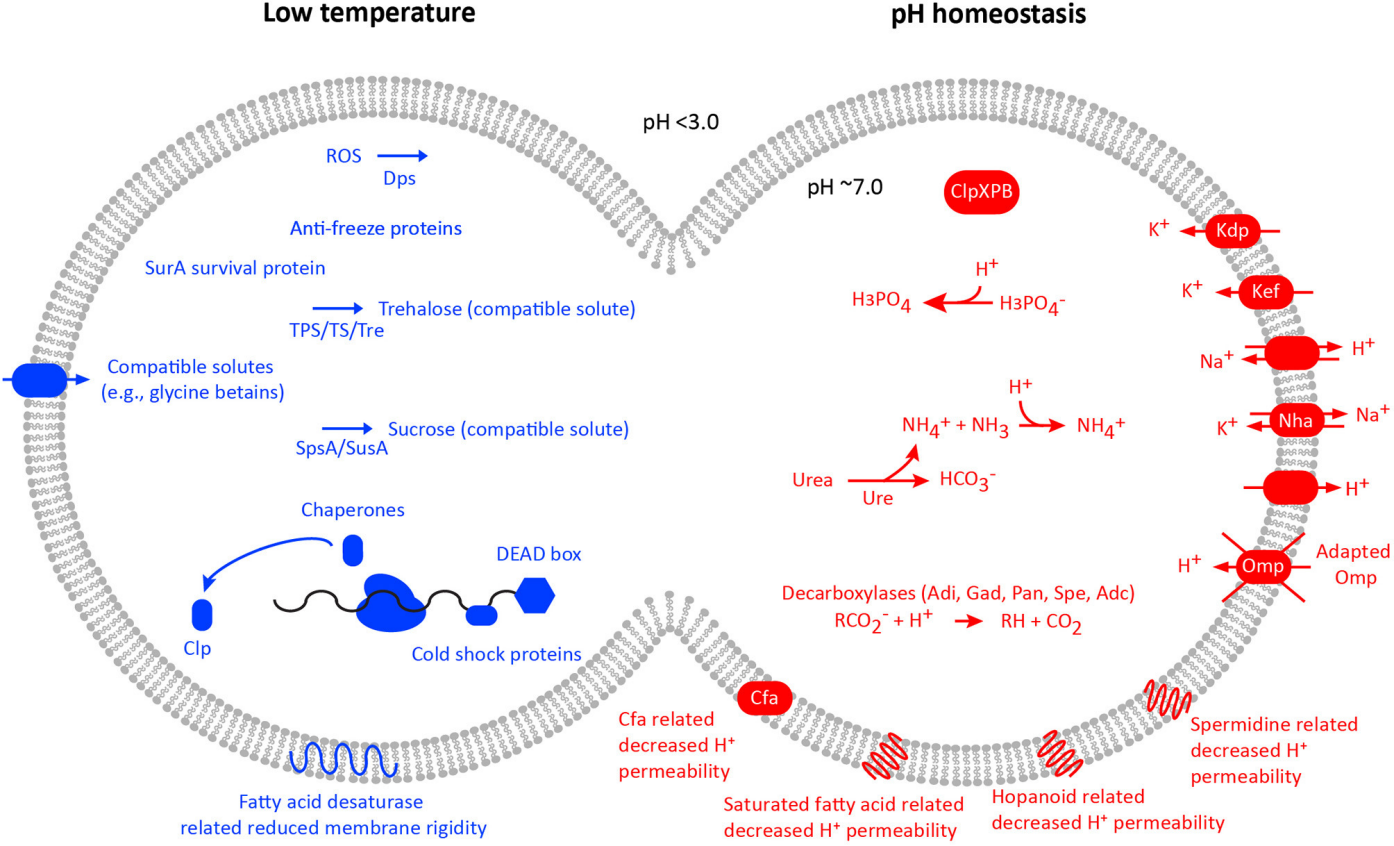
Acidophile and low-temperature adaptations for selected genomes based upon complete genomes and type strains of the available species. Data used during the preparation of the figure were drawn from the AciDB database of acidophilic organisms (Neira et al., 2020).

Microbial cells that can tolerate multiple physiochemical extremes are polyextremophiles, and they maintain cell homeostasis despite the pressures exerted on the cell (Figure 3). Low-temperature acidophiles exist in such environments where adaptation to both cold and acid stress can be different from the adaptation mechanisms commonly observed to either stress alone (Figure 4). While eurypsychrophiles are discussed in this study, there are no known stenopsychrophile acidophiles, and we hypothesize that the nature of some adaptations may have inhibited the evolution of acidophiles that exclusively grow below 15°C.

The adaptations to an acidophilic lifestyle identified in *At. ferrivorans* SS3 include a potassium transporting ATPase suggested to be involved in the inside positive (inversed) membrane potential and a P-type ATPase proton efflux pump that was not present in an *At. ferrivorans* MAG from a low-temperature AMD stream (González et al., 2014). In addition, the *At. ferrivorans* strain YL15 genome contains genes for the inversed membrane potential-related *kdp* potassium uptake system and a sodium:potassium antiporter; the membrane hopanoid squalene synthesis and associated genes *hpnAIJKNHM*; proton-consuming cytoplasmic buffering systems *adi* and *gadB*; and the acid resistance *clpXP* APT-dependent Clp protease genes (Peng et al., 2017). Furthermore, *At. ferrivorans* XJFY6S-08 contains *kdpDEABC* genes for the potassium uptake system; the *nhaA* sodium/proton antiporter; a *shc* squalene-hopene/tetraprenyl-beta-curcumene cyclase; *clpXPB* Clp protease; and proton-consuming *speA* arginine decarboxylase and *gadABC* glutamate decarboxylase (Zhao et al., 2021). Finally, a decreased proton permeability *via* an increased saturated fatty acid composition was most pronounced in *At. ferrivorans* growth at pH 1.5 (Mykytczuk et al., 2010a). *Ferrovum* strain JA12 adaptations to acidic pH include a Kef-type K^+^ transport system and a K^+^ transporter; *cfa* cyclopropane-fatty-acyl-phospholipid synthase membrane protein; cytoplasmic buffering systems including *adi* arginine decarboxylase, *panD* phosphatidylserine decarboxylase, and the *ureABCDEFGHJ* urease system; and spermidine synthase that inhibits proton influx *via* porins (Ullrich et al., 2016b). *Ferrovum* strain JA12 RNA transcripts in response to growth on ferrous iron showed maintenance of pH homeostasis including for the cyclopropane-fatty-acyl-phospholipid synthase, the Kef-type K^+^ transport system, a K^+^ transporter, and cytoplasmic buffering (Ullrich et al., 2018). In addition, “*Ferrovum*” strains P3G and Z-31 harbor the *kdpABCDE* K^+^-transporting ATPase and the *nhaA* sodium/proton antiporter (Ullrich et al., 2016a). Finally and with the above-mentioned caveat regarding average environmental pH and temperatures, RNA transcripts assigned to the “*Ferrovum*” P3G MAG from an AMD site included the *kdpDE* signal transport system, a sodium:proton antiporter, and arginine decarboxylase (Hua et al., 2015). An additional AMD metagenome *Ca*. Gallionella acididurans MAG encoded the *kdpABC* K^+^-transporting ATPase (Kadnikov et al., 2016), while a community analysis of Chinese low-grade copper tailings also assigned coding sequences to *kdpABC*, a voltage-gated potassium channel, and proton antiporters assigned to among other MAGs, *At. ferrivorans* (Zhang et al., 2016). Finally, community RNA transcript sequencing of the Finnish low-temperature boreal acid sulfate soil (Högfors-Rönnholm et al., 2022) revealed transcripts at the metal sulfide oxidation front coding for both “first line of defense” and “second line of defense” acid resistance systems including potassium transporters and components of the arginine-dependent acid resistance system (González-Rosales et al., 2022).

Low temperature adaptations identified in *At. ferrivorans* include production of the compatible solute trehalose (Liljeqvist et al., 2011b) that that has also been demonstrated to have a function in e.g., acidophile osmotic stress tolerance (Galleguillos et al., 2018). However, it was argued that the multiple trehalose synthesis pathways in a *At. ferrivorans* MAG from a low-temperature AMD metagenome compared to mesophilic *Acidithiobacillus* spp. reflected its greater importance for growth at low temperatures (Liljeqvist et al., 2015). In addition, the AMD stream MAGs of the dominant populations contain further adaptations to the low temperature including cold-shock proteins and an anti-freeze protein (Liljeqvist et al., 2015). The *At. ferrivorans* CF27 genome also contains genes for adaptation to low temperatures including production of the compatible solutes trehalose and sucrose, cold shock proteins, chaperones, and membrane adaptations (Tran et al., 2017). Cold adaptation genes were also present in *At. ferrivorans* PQ33 including DEAD/H-box RNA helicases; fatty acid desaturases genes that increase membrane fluidity; trehalose synthase genes; and protein folding peptidyl-prolyl cis/trans isomerases (Ccorahua-Santo et al., 2017). Finally, *At. ferrivorans* XJFY6S-08 contains genes for low temperature such as chaperones, e.g., *groEL/ES* and trigger factor with peptidyl-prolyl cis/trans isomerase activity; five trehalose synthase pathways; and cold shock proteins (Zhao et al., 2021). However, the growth of *At. ferrivorans* SS3 at 8°C showed few RNA transcripts related to cold stress supporting its assignment as a eurypsychrophile (Christel et al., 2016b). In contrast, *At. ferrivorans* strain YL15 RNA transcript analysis identified systems for growth at low temperatures including membrane transport and energy metabolism along with *dps* gene transcripts for protection against oxidative stress (Peng et al., 2017). Proteomic comparisons of *At. ferrivorans* D6 strain to mesophilic strains of *At. ferrooxidans* grown near their lower temperature limits of 5°C and 15°C, respectively, revealed that both organisms shared increased levels of certain stress-related proteins (survival protein SurA, trigger factor, and antioxidant proteins) in response to colder temperatures (Mykytczuk et al., 2011a). However, the eurypsychrophilic strain D6 revealed upregulation of a significantly higher number of proteins related to membrane transport and pH homeostasis (e.g., ABC transporters and the MotA-TolQ-ExbB proton channel family protein) and membrane structure (e.g., PAP2 phosphatidic acid phosphatase, glycosyl transferase, and capsule polysaccharide export protein) compared to the mesophilic *At. ferrooxidans* (Mykytczuk et al., 2011a). Membrane adaptation in these eurypsychrophilic *At. ferrivorans* strains was notably different from mesophilic strains of *At. ferrooxidans* with an increase in desaturated fatty acids (16:1 w6c and 17:1 w8c) but a more rigid cytoplasmic membrane with a broader membrane phase transition temperature range in response to cold temperatures (Mykytczuk et al., 2010b). Finally, *At. ferrivorans* strain YL15 transcripts related to energy metabolism at colder temperatures also showed a 4 fold increase in rusticyanin production compared to growth at warmer temperatures. Similarly, Peng et al. (2017) observed increased expression of iron oxidation (*rusA* and *cycA1*) and sulfur oxidation genes (*hdrA, cyoC1*, and *doxDA*) of *At. ferrivorans* YL15 at 6°C compared to growth at 28°C. Both low temperature *At. ferrooxidans* strains PG05 and MC2.2 have draft genome sequences from which an analysis of the nine available *At. ferrooxidans* genomes showed, e.g., higher gene frequencies for genes encoding cold tolerance compared to the type strain (Muñoz-Villagrán et al., 2022). These included genes for the compatible solute trehalose that has several functions including cold tolerance, a transporter for a further compatible solute glycine betaine, fatty acid desaturases, and hopanoid biosynthesis. In addition, genes coding for low-temperature-induced oxidative stress were particularly abundant in strain MC2.2.

## Biotechnology

Eurypsychrophilic acidophiles have a niche for potential applications in biotechnology that differentiates them from applications under solely low temperature or acidic conditions. The clearest case for the application of eurypsychrophilic acidophiles is in metal dissolution and bioremediation of mining processes and wastewaters in boreal environments or at high altitudes. Other potential applications include targeting these populations for enzymes that can be used for other, non-mining applications.

### Biomining and remediation

Bioheaps for nickel, zinc, cobalt, and copper recovery from a low-grade black schist ore in North-Eastern Finland have been in operation since 2008 at the Terrafame mine [originally Talvivaara Mining Company; (Riekkola-Vanhanen, 2010, 2013)]. Biomining of the metals was successful despite the average temperatures in winter ranging between zero to −20°C (with extreme lows of −30°C) as exergonic reactions during principally pyrrhotite oxidation resulted in the inner areas of the bioheap reaching 90°C (Halinen et al., 2012). Eurypsychrophilic acidophiles would have been important during bioheap initiation before the core had increased in temperature and in the niche temperature zone between the inner, hot bioheap and the extremely cold surface during the winter months. Early laboratory column studies of the black schist showed relatively high rates and yields at 5°C compared to 21°C (Puhakka et al., 2007). Molecular identification of the column microbial communities enriched at 5°C from mine site waters identified strains similar to *At. ferrooxidans* along with *At. thiooxidans* (Puhakka et al., 2007). However, at this time, strains now known to be *At. ferrivorans* were typically classified as *At. ferrooxidans*, and further work with the black schist ore suggested that *At. ferrooxidans* A7 is eurypsychrophilic (Dopson et al., 2007). Further laboratory tests of column bioleaching at 5°C vs. 30°C showed a small decrease in metal dissolution from pyrite, pyrite/arsenopyrite, and chalcopyrite concentrates when *At. ferrivorans* SS3 was used compared to *At*.*ferrooxidans*^T^ (Dopson et al., 2007). Chalcopyrite bioleaching was also investigated using a consortium enriched from AMD dominated by *Acidithiobacillus* spp. and *Sulfobacillus* spp. that outcompeted *At. ferrivorans* strain YL15 at 6°C (Peng et al., 2021). Finally, the accumulation of solid sulfur during chalcopyrite bioleaching was observed that was explained by decreased gene expression of genes coding for ISC oxidation in *At. ferrivorans* SS3 (Liljeqvist et al., 2013).

Another advantage of the activity of eurypsychrophilic acidophiles in bioheap applications, with for example *At. ferrivorans* strains, could be gained through their initiation of the oxidation process while limiting mineral passivation. In laboratory tests both in liquid media and on various sulfide tailings, the production of jarosite was greatly reduced at 5°C, decreasing the rate of mineral passivation and allowing Fe^3+^ concentrations to rise within the aqueous phase compared to higher incubation temperatures (Leduc et al., 1993; Mykytczuk et al., 2010b). In stirred bioreactor systems, it is unlikely that colder temperatures would be favorable or possible to achieve given the exothermic reactions; however, retaining ferric iron in solution could allow for the recycling of the oxidant. Recently, Peng et al. (2017) tested *At. ferrivorans* strain YL15 in chalcopyrite leaching in shake flasks at 6°C and observed no passivating layers on the mineral surface although corrosion was evident by scanning electron microscopy. Finally, all the tested *At. ferriphilus* strains catalyzed pyrite dissolution (Falagan and Johnson, 2016).

As detailed above, eurypsychrophilic acidophiles are often identified in low-temperature AMD environments (e.g., Escobar et al., 2010), and they can be exploited for the bioremediation of these contaminated waters. For example, room-temperature ferrous iron-oxidizing bioreactors were used to treat AMD that over time became dominated by a community including “*F. myxofaciens*” and *At. ferrivorans* (Jones and Johnson, 2016). Low temperature, passive systems such as wetlands, and active treatments to oxidize ferrous to ferric iron have also been studied [reviewed in Kaksonen et al. (2008)]. An example of low-temperature bioremediation is schwertmannite and jarosite production using “*F. myxofaciens*” EHS6 to remove iron- and sulfate from mine water at 14.5°C (Hedrich et al., 2011). A second example is the removal of ISCs from tailings water down to 5°C to 6°C with a mixed consortium dominated by *At. ferrivorans* (Liljeqvist et al., 2011a). Finally, coal desulfurization has been carried out with an *At. ferrivorans* strain (Aytar et al., 2013).

## Conjecture on the survival of acidophilic eurypsychrophiles during snowball earth events

During its history, Earth is thought to have undergone several episodes of almost complete ice and snow-coverage that are known as Snowball Earth events (Kirschvink et al., 2000). Although mesophilic and thermophilic microorganisms could have survived such conditions in a limited number of warmer niches under the ice (e.g., thermal fields), psychrophiles potentially had an opportunity of surviving in a wider range of cold environments during these time periods. Of particular interest is the possibility that some acidophilic eurypsychrophiles might be especially adapted to survive cold temperatures by virtue of their ability to raise the temperature of their surroundings by catalyzing exothermic iron and sulfur oxidation reactions (Weast, 1978/79; Mraw and Naas, 1979). In addition, the presence of sulfuric acid lowers the freezing temperature of water (Gable et al., 1950) which could allow acidophiles to survive at sub-zero temperatures with the use of cellular cryoprotectants potentially enhancing that effect.

## Analogs for life detection on planets and moons

Earth's extremophiles and their habitats are being investigated as analogs in the search for potentially habitable environments on exoplanets and moons (Martins et al., 2017; Merino et al., 2019; Carré et al., 2022). Mars, Enceladus (moon of Saturn), and Europa (moon of Jupiter) are candidates to harbor present or past life. Mars likely had a large water ocean in its earlier history, but it has subsequently dried out at least on its surface. As it dried out, lacustrine evaporates were created some of which are thought to have been acidic (Tosca et al., 2005). Eventually, the surface of Mars became hyperarid, extremely cold, and subjected to high levels of UV radiation. Today, water only remains in the subsurface cryosphere (Carr and Head, 2019) and under the south polar ice cap (Orosei et al., 2018).

Several sites around the world are being studied as analogs for ancient and modern Martian sites that could have harbored (or still do) acidophilic eurypsychrophiles (Fairén et al., 2010). These include acidic saline lakes in Western Australia (Benison and Bowen, 2006) and cold acidic lakes in the Altiplano of northern Chile that are also subjected to elevated levels of UV radiation because of the high altitude (Risacher et al., 2002; Demergasso et al., 2010). Terrestrial analog sites for potential life under ice or on the cold surface include Vostok ice in Antarctica (Price, 2000); arctic mineral assemblages in northern Greenland (Langdahl and Ingvorsen, 1997) and the Antarctic (Dold et al., 2013; Garcia-Lopez and Cid, 2017); and in sulfidic caves across the globe (Hose et al., 2000; Vlasceanu et al., 2000; Polyak and Provencio, 2001; Engel et al., 2004; Galdenzi et al., 2008; Jones et al., 2014; D'angeli et al., 2019). Metagenomic analyses of several cave and low-temperature environments revealed the presence of known extreme acidophiles (Langdahl and Ingvorsen, 1997; Vlasceanu et al., 2000) but none have been tested in the laboratory for low-temperature growth.

Both Enceladus and Europa are thought to contain water oceans under kilometers of ice (Carr et al., 1998; Collins and Goodman, 2007) that are kept liquid by ocean floor hydrothermal activity (Waite et al., 2017) or tidal forces (McKay et al., 2012). The ocean of Enceladus is thought to be alkaline (Glein et al., 2015) but there may be regions on the ocean floor that are more acidic such as are found close to terrestrial black smokers. The ocean of Europa may be acidic (Pasek and Greenberg, 2012) although this is controversial (Kargel et al., 2000; Russell et al., 2014). In either case, pH is likely to vary along strong geochemical gradients within the oceans on the icy moons displaying the potential for strongly alkaline to acidic habitats (McKay et al., 2012; Russell et al., 2014). Some of the terrestrial cold, acidic environments mentioned in the previous paragraph are being investigated as analogs for the oceans of Enceladus and Europa (Marion et al., 2003).

## Moving forward

During the preparation of this manuscript, several environmental studies lacked the appropriate sample metadata that would have allowed the unequivocal identification of the environment as both acidic and cold. The importance of such data cannot be overly stressed. For example, in sulfidic cave studies where the air temperature deep inside the cave might be assumed to be cold, the actual temperature of the sample might be higher due to exothermic chemical and biological sulfide oxidation reactions. This potential creation of a “warm niche” that may promote the survival of co-habituating microorganisms (e.g., in biofilms in bioleaching heaps and environmental samples) with sulfide-oxidizing microbes is largely uninvestigated in laboratory simulations. A second lacuna in our knowledge that became evident was the lack of, e.g., RNA transcript-based support for the response to concurrent low temperature and acidic pH that should be a target for future studies.

Low-temperature and acidic environments have received less attention than their counterparts inhabiting higher temperatures, potentially as they are less easily accessed, e.g., at high altitudes or in Antarctica. Furthermore, although not covered in this review due to typically being inhabited by moderate acidophiles, arctic tundra environments are becoming more prominent in relation to thawing of permafrost due to climate change (Kwon et al., 2019). In consequence, this valuable niche remains untapped for, e.g., biotechnological applications plus understanding future climate and highlights the need for further investigation.

A notable gap in low-temperature acidophile microbiology is the lack of a true “stenopsychrophile” (i.e., growth at a narrow temperature range below 15°C). Potential environments to discover psychrophilic acidophiles are dark oligotrophic volcanic ecosystems, e.g., on Mount Erebus, Antarctica (Tebo et al., 2015) and Black Smokers (Han et al., 2018). Black Smokers are hydrothermal vents found on the ocean floor where spreading ridges occur as the result of either the moving apart of tectonic plates (Douville et al., 2002) or where tectonic plates are moving toward one another creating back-arc basins (Mottl et al., 2011; Reeves et al., 2011). The vents form when cold seawater is heated by underlying magma and re-emerges forming chimneys that spew out usually acidic (< pH 3.5) black fluid laden with sulfide mineral precipitates. However, the venting fluid can be extremely hot with temperatures reaching over 370°C (Von Damm, 1990) with significant differences in the chemical composition due to the nature of the underlying magma. For example, if the magma is hosted by basalt, the venting fluid is H_2_S rich, H_2_ poor, and less reducing, whereas if it is hosted by ultramafic rock, it tends to be H_2_, Fe, and methane-rich and more reducing. These geological characteristics lead to variations in chimney composition and structure and the nature of surrounding sea-floor precipitates that, in turn, shape microbial community structure. Black smokers might seem like a poor place to search for eurypsychrophilic acidophiles, and indeed, thermophilic microbes abound in vent metagenome analyses. However, some vents are cool (< 20°C), and since vents are surrounded by seawater of ~2°C, there are potentially habitats that could be occupied by eurypsychrophilic acidophiles (Adam et al., 2020). Metagenomic analyses have been carried out of several vent sites including the outside of chimneys with recorded temperatures of < 20°C and in nearby seafloor precipitates (2°C). In these studies, populations have been detected that are phylogenetically related to known acidophile phylotypes. However, the phylogenies do not provide sufficient resolution to identify known eurypsychrophilic acidophiles at the species level. In addition, no laboratory work has been carried out to experimentally determine if eurypsychrophiles exist on or close to venting chimneys (Nercessian et al., 2005; Flores et al., 2011; Ding et al., 2017; Hager et al., 2017) or if they are capable of growth at < 15°C. These locations are especially interesting targets to search because the isolated nature of vent fields could promote allopatric speciation, potentially increasing the degree of microbial diversity.

## Author contributions

MD conceived the study. All authors contributed to data collection, analysis, and manuscript preparation. All authors read and approved the final manuscript.
